# Development of a Bendable Outsole Biaxial Ground Reaction Force Measurement System

**DOI:** 10.3390/s19112641

**Published:** 2019-06-11

**Authors:** Junghoon Park, Sangjoon Jonathan Kim, Youngjin Na, Yeongjin Kim, Jung Kim

**Affiliations:** 1 Department of Mechanical Engineering, Korea Advanced Institute of Science and Technology (KAIST), Daejeon 34141, Korea; junghoon.park@kaist.ac.kr (J.P.); sangjoon.j.kim@kaist.ac.kr (S.J.K.); 2 Division of Mechanical Systems Engineering at Sookmyung Women’s University, Seoul 04312, Korea; yjna@sookmyung.ac.kr; 3 Division of Thermal and Fluids Science, Institute for Computational Science, Faculty of Electrical and Electronics Engineering, Ton Duc Thang University, Ho Chi Minh City 758307, Vietnam; Ykim@tdtu.edu.vn; 4 Department of Mechanical Engineering, Incheon National University, Incheon 22012, Korea; ykim@inu.ac.kr

**Keywords:** ground reaction force, biaxial force sensor, reflective photomicrosensor, outsole system, forefoot bending stiffness

## Abstract

Wearable ground reaction force (GRF) measurement systems make it possible to measure the GRF in any environment, unlike a commercial force plate. When performing kinetic analysis with the GRF, measurement of multiaxial GRF is important for evaluating forward and lateral motion during natural gait. In this paper, we propose a bendable GRF measurement system that can measure biaxial (vertical and anterior-posterior) GRF without interrupting the natural gait. Eight custom small biaxial force sensors based on an optical sensing mechanism were installed in the proposed system. The interference between two axes on the custom sensor was minimized by the independent application of a cantilever structure for the two axes, and the hysteresis and repeatability of the custom sensor were investigated. After developing the system by the installation of force sensors, we found that the degree of flexibility of the developed system was comparable to that of regular shoes by investigating the forefoot bending stiffness. Finally, we compared vertical GRF (vGRF) and anterior-posterior GRF (apGRF) measured from the developed system and force plate at the same time when the six subjects walked, ran, and jumped on the force plate to evaluate the performance of the GRF measurement system.

## 1. Introduction

The measurement of ground reaction force (GRF) is a representative kinetic analysis method in biomechanics, and the GRF measurement has been widely used in the diagnosis of abnormal gaits, control of exoskeleton, and evaluation of athletics’ performance [1,2,3,4]. A stationary force plate has been primarily used to measure the GRF [5,6], but this method can be used only in a controlled environment, and the awareness of stepping on the correct position of the force plate can hinder one’s natural gait [7,8]. To overcome the drawbacks of the force plate, many efforts have been focused on the development of wearable GRF measurement systems. A wearable system can be used in various environments while measuring the GRF during natural walking and can be used to analyze various gait parameters, such as gait speed and stride length, among others, without distortion due to hindering the natural gait. 

Wearable GRF measurement systems can be categorized as either the insole type or outsole type. The insole type system is developed by attaching thin and flexible force sensors onto the sole, which does not hinder normal gait [9,10,11]. GRF can be measured during ski jumping or a squat since it can provide the natural gait. Although the insole type system ensures flexibility for natural gait, they cannot simultaneously measure multiaxial GRFs and are difficult to use over a long period of time due to their low durability [12,13]. To estimate multiaxial GRF with the insole type system, some studies have tried to combine the kinematic data by using an inertia measurement unit (IMU) with the kinetic data from GRF [14,15]. However, it has low estimation accuracy or high accuracy only for certain repetitive actions. Unlike the insole type system, the outsole type system can measure multiaxial GRFs with rigid multiaxial force sensors and produce more accurate GRFs than the insole type system because the sensors have direct contact with the ground [16,17,18,19,20]. Despite the advantage of the outsole system, the flexibility of the outsole type system is low because of the large force sensors installed at the front and rear of the foot [16,17,18,19]. Because these rigid objects are located in the outsole, unlike regular shoes, natural walking can be hindered due to reduced flexibility [20]. To improve the flexibility, a force sensor that is smaller than previous force sensors must be developed, and these force sensors must be installed in the optimal locations to measure the accurate GRF. In addition to sensor size issues, previous studies have not considered the structure of the outsole to supply flexibility similar to that of regular shoes in which the outsole has adequate flexibility to ensure user wearability. To evaluate the flexibility of shoes, researchers have considered the forefoot bending stiffness, which indicates the stiffness of the metatarsophalangeal (MTP) joint [21,22]. The forefoot bending stiffness of the footwear is important because it changes the function of the MTP joint and other mechanics of the lower limb joints [23]. From this perspective, the flexibility of the outsole system can be improved by changing the outsole structure to make the forefoot bending stiffness of the system more similar to that of regular shoes.

In this study, we developed a small biaxial force sensor and installed the developed force sensors at the optimal locations to improve the flexibility of the outsole type GRF measurement system. A custom small biaxial force sensor was built based on a previously developed bulky biaxial force sensor that was designed using reflective photomicrosensors [24,25]. The custom force sensor can measure both the vertical GRF (vGRF) and anterior-posterior GRF (apGRF). To validate the performance of the developed sensor, various sensor characteristics, such as the hysteresis, repeatability, and cross sensitivity, were investigated. In addition, a GRF measurement system with a flexible outsole structure made using a 3D printed rubber frame was designed to increase flexibility, and the forefoot bending stiffness of the system was investigated through comparison with that of regular shoes.

Section 2 introduces the design of the custom force sensor and a GRF measurement system, and Section 3 focuses on the experiments results, such as the characteristics of the developed sensors and the forefoot bending stiffness of the developed system. In addition, we conducted the experiments to identify whether the system could hinder the natural gait or not and could be used to measure GRF during walking, running, and jumping. Finally, we offer concluding remarks in Section 4.

## 2. Methods and Materials

### 2.1. Biaxial Force Sensor Design

This section describes the design and characteristics of the proposed force sensor built using reflective photomicrosensors. Our custom force sensor can simultaneously measure the normal and shear forces with a high load capacity (1000 N for the normal force and 300 N for the shear force). The maximum GRFs in the vertical direction, anterior-posterior direction, and medial direction are 120%, 20%, and 5% of the body weight, respectively [26]. Because the maximum GRF in the medial-lateral direction is smaller than the other two GRF components, we considered only the GRFs in the vertical and anterior-posterior directions. Using the Pedar insole, which is a typical insole type GRF measurement system, Pu et al. determined how the size, number, and location of sensors influence the estimation of the total plantar force with a limited number of sensors [27], and concluded that a 30 mm × 30 mm sensor layout with four (2 × 2) or nine (3 × 3) of the 99 conductive force sensors yielded the best plantar pressure estimation performance. Therefore, we chose this size for our proposed sensor. The height of the sensor was set as low as possible considering the height of the sensor in the existing outsole system [20].

**Figure 1:**
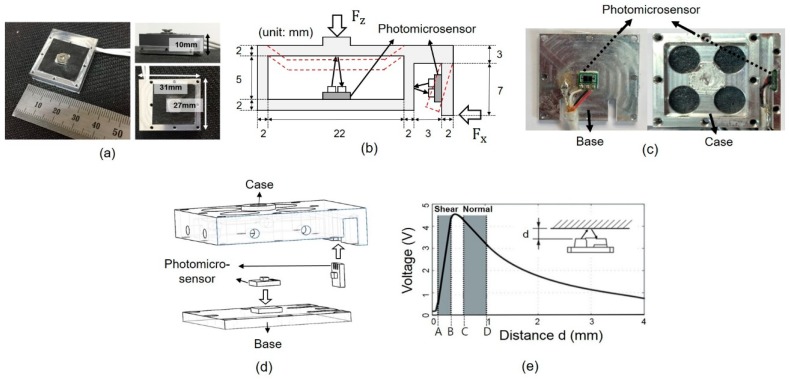
Design of the custom biaxial force sensor. (**a**) Size of the custom force sensor. (**b**) Measurement principle of the force sensor and detailed dimensions of the sensor. (**c**) The location of the printed circuit board (PCB) of the photomicrosensor. (**d**) Assembly diagram of the force sensor. (**e**) Voltage output according to the displacement of the photomicrosensor and the sensing range for shear (A–B) and normal (C–D) directions.

Figure 1a indicates the overall design of our custom force sensor, which had a weight of 12 g, and the frames of the sensor were made of aluminum 6061-T6. The main parts of the custom force sensors were the case, the base, and two photomicrosensors. The case and base were manufactured by a computerized numerically controlled machine tool (CNC). The sensor was developed using two reflective photomicrosensors (EESY199, Omron, Tokyo, Japan) as shown in Figure 1b. Two photomicrosensors were attached at the bottom of the base and side of the case shown in Figure 1c. 

Each photomicrosensor was attached to the case and the base by adhesive (Loctite 401, Loctite, Duesseldorf, Germany) as shown in Figure 1d. When the forces were loaded on the top ($F_{z}$) and the edge ($F_{x}$) of the case, the case deformed as shown by the red dotted line in Figure 1b and the distance between the photomicrosensor and frame began to change. The distance between the photomicrosensor and frame was measured by the intensity of light from the photomicrosensor. The reflective photomicrosensor converts the deformation change to the output voltage change. The deformation ranges in the shear direction varied between A and B (approximately 0.2 mm and 0.4 mm), and those in the normal direction varied between C and D (approximately 0.85 mm and 1 mm), as shown in Figure 1e. To measure the large force with a high resolution, the deformation range for the normal direction was larger than the range for the shear direction. The relationship between distance and voltage was linear in the range A–B for the shear direction and quadratic in the range C–D for the normal direction. In addition, the relationship between the force loaded on the plate and the distance change was linear because of the intrinsic characteristics of the fixed beam structure for the normal direction and the cantilever beam structure for the shear direction. Therefore, the relationships between force and voltage were linear for the shear direction and quadratic for the normal direction, as expressed in Equation (1):

(1) $$F_{z}=a_{1}V_{z}^{2}+a_{2}V_{z}+a_{3},$$

$$F_{x}=b_{1}V_{x}+b_{2}$$

where *Vz* and *Vx* denote the voltage measured by the reflective photomicrosensors for the normal and shear directions, respectively, *Fz* and *Fx* indicate the forces loaded on the sensor, and *ai* and *bi* are the coefficients of each sensor. Calibration was performed for each sensor in the normal and shear directions according to Equation (1). 

The advantage of our custom force sensor with reflective photomicrosensors is that the voltage output can be measured without any additional amplifiers, unlike a strain gauge type load cell. The power consumption was 100 mW (5 V–20 mA) for each sensor, which was sufficiently low to use a small Li-ion battery as the power supply.

### 2.2. GRF Measurement System Design

**Figure 2:**
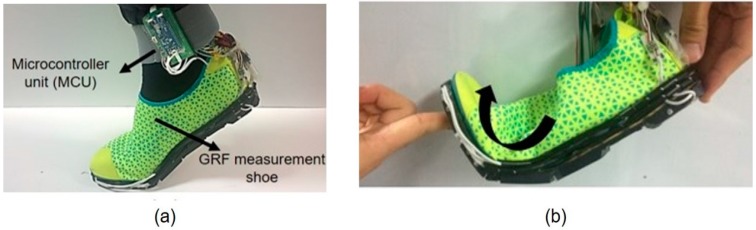
Bendable outsole biaxial ground reaction force measurement system. (**a**) Developed system connected to the microcontroller with a Bluetooth module. (**b**) Developed system with a bendable design.

**Figure 3:**
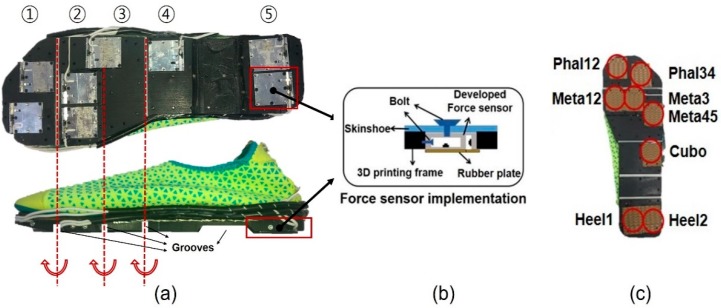
Design of the developed GRF measurement system. (**a**) Installation of the 3D printed rubber frame, in which the four grooves cause the system to bend only upward. (**b**) The method used to install the developed force sensor under a water shoe (Skinfit wave, BALLOP, Seoul, South Korea). (**c**) The locations of the eight developed force sensors in the system.

Figure 2a shows the GRF measurement system developed using the custom force sensors. It is possible to develop the system to not hinder the natural gait because the system can be bent upward easily as shown in Figure 2b. A black rubber frame (Tango black plus FLX 980, Stratasys, Rehoboth, Israel) built using a 3D printer was attached under the thin-sole shoe to support the force sensors, as shown in Figure 3a. The frame ensured that the thin-sole shoe bends only in one direction because the grooves ensure that the entire frame bends in one direction. Thus, the metatarsal joint allowed bending only in the upward direction during walking, similar to regular shoes. The thickness of the frame was less than the height of the custom force sensor to load the entire GRF on the force sensors. The force sensors were fastened under the thin-sole shoe and to the sides of the case of the force sensor using bolts, except the side with the photomicrosensor measuring the shear force. Thin rubber plates were attached to the bottom of the force sensors to increase the friction between the sensor and the ground, which resulted in a higher sensitivity for the shear force. 

When installing the force sensors in the system, we considered the bone and muscle structure of the foot. The human foot consists of 26 bones [28], and the bone and muscle structures of the human foot vary from person to person. The plantar foot pressure differs depending on sex, age, body weight, foot size, and even various movements of the human subject [29,30]. In order to measure the GRF for various motions regardless of the foot structure, a small force sensor and the area sensors covered should be enough to measure GRF accurately [31]. However, the small force sensor that can measure the biaxial forces with a high capacity and high repeatability cannot be easily constructed. Therefore, the proper numbers and locations of the force sensors must be determined to more accurately measure the GRF with a limited number of sensors. We decided the locations and number of sensors considering the previous study, which measured foot pressure distribution (FPD) by using shoe insoles with 99 capacitive sensors during normal walking in healthy young and elderly subjects [32]. Since the experiment was conducted on healthy people in the existing study, the developed system is also applicable to healthy people. From the result of the previous research, eight regions were chosen for the force sensor locations as shown in Figure 3c. These regions covered an area in contact with the toe bones (phalanges), metatarsal bones, cuboid bone, and heel bone (calcaneus), thus these sensors were denoted as Heel1, Heel2, Cubo, Meta12, Meta3, Meta45, Phal12, and Phal34. For the heel area, two force sensors were installed because the sensors have to cover the average heel contact area with the ground during walking, which is twice as large as the surface area covered by one custom force sensor [33]. Although the GRF concentrated point was between two sensors at the heel, the signal of each sensor occurs simultaneously and the GRF at the heel is the sum of the force measured by each sensor. Moreover, in order to measure GRF during running and jumping, the voltage signal has to be transmitted through wireless communication to make the subject comfortable. In addition, it is better for the data acquisition system to have large bit number (12-bit) to increase the resolution of the force sensor. Therefore, a microcontroller unit (MCU) with a large bit number and Bluetooth communication system was designed as shown in Figure 2a. The central processing unit (CPU) of the developed MCU was ARM^®^ Cortex^®^-M4/192KB with reference STM32F405RGT6. The power of MCU can be supplied by Li-ion battery (1.8~3.6V) and the total weight with the battery was lower than 100 g.

## 3. Experiment Results and Discussion

### 3.1. Experiments for Biaxial Force Sensor

In this section, we conducted finite element analysis to investigate the deformation of the sensor frames and performed the experiment to investigate the sensor characteristics, such as hysteresis, repeatability, and cross sensitivity.

#### 3.1.1. Finite Element (FE) Analysis 

**Figure 4:**
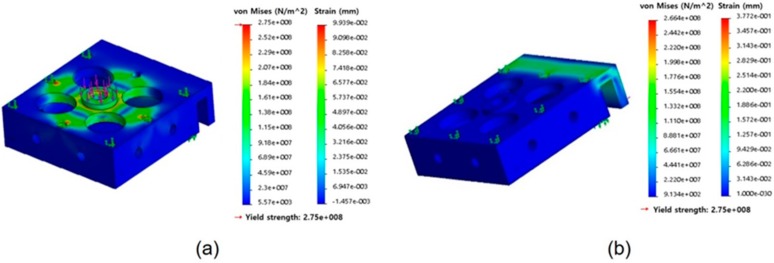
Finite element (FE) analysis results for the developed force sensor. (**a**) FE analysis of the normal direction and (**b**) FE analysis of the shear direction.

In order to verify whether the sensor frames can withstand the force when the deformation was 1000 N of the normal direction and 300 N of the shear direction, we conducted the FE simulation (ABAQUS/Standard 6.5.1, SIMULIA, Providence, Rhode Island, USA). The FE model consisted of 59,077 nodes with 36,515 elements. The deformation ranges of the sensor frame were verified using FE simulation, as shown in Figure 4a,b. The von Mises analysis shows that deformation occurs within the yield stress for the maximum force applied in the vertical and horizontal directions. In other words, it means that the sensor can endure 1000 N for the normal direction and 300 N for the shear direction. 

#### 3.1.2. Sensor Characteristics

**Figure 5:**
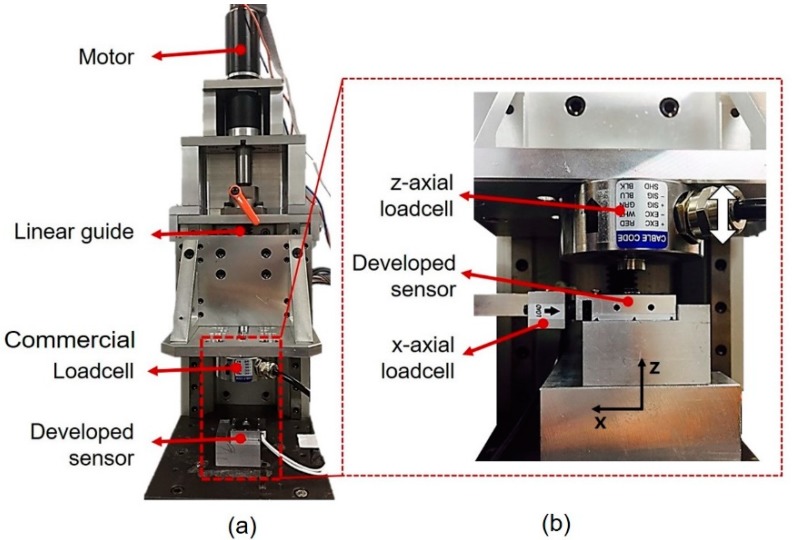
Experimental setup used to analyze the sensor characteristics. (**a**) An indenter used to analyze hysteresis, repeatability, and cross sensitivity. (**b**) Additional setup for the cross sensitivity analysis of the normal direction to the shear direction and the cross sensitivity analysis of the shear direction to the normal direction.

We investigated the calibration results, hysteresis, repeatability, and cross sensitivity of our sensor. The indenter setup consisted of a linear guide, a DC motor (RE40 148877, Maxon Motor, Switzerland), and a gearbox (GP42 203125, Maxon Motor, Sachseln, Switzerland) and pressed the custom sensor periodically, as shown in Figure 5a. The custom sensor was attached to the bottom plate of the indenter, and a commercial load cell (MNC-200, CAS Co., Ltd., Seoul, South Korea) was located under the top plate, which can be moved up and down. The maximum load capacity of the commercial load cell was 2000 N. The output voltages of the commercial load cell and custom sensor were acquired simultaneously from a data acquisition board (Q8, Quanser Co., Ltd., Markham, ON, Canada) with a 1 kHz sampling rate. For cross sensitivity measurement, an additional commercial load cell (651AL, Ktoyo Co. Ltd., Seoul, South Korea) with a maximum load capacity of 200 N was installed, as shown in Figure 5b. When the force was applied along *z*-axis, the *x*-axis commercial load cell measured the force generated by the deformation in the shear direction. In other words, the *z*-axis load cell measured the force of the deformation in the normal direction, while the other load cell measured the *x*-axis force.

**Figure 6:**
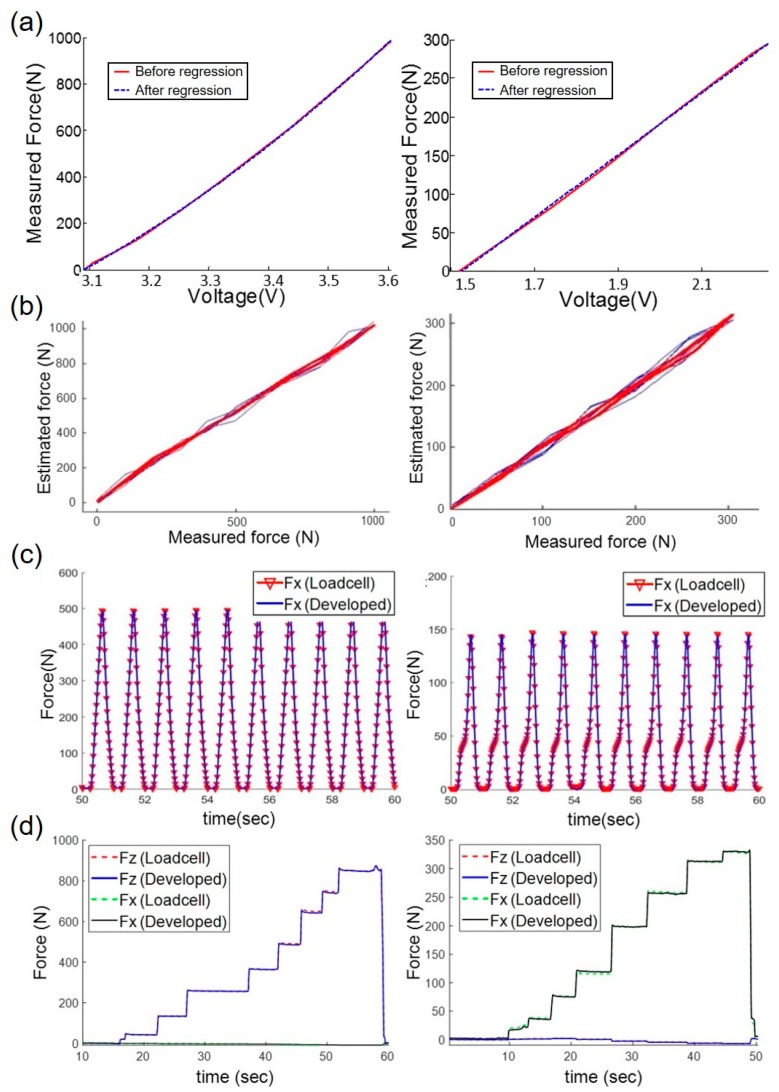
Sensor characterizations. (**a**) Calibration results of the developed force sensor for the normal (left) and shear direction (right). (**b**) Hysteresis of the developed force sensor with the load cell for the normal (left) and shear direction (right). (**c**) Response of the developed force sensor with a repeated load for the normal (left) and shear directions (right). (**d**) Response of the developed force sensor for the normal direction when the shear force is loaded (left) and for the shear direction when the normal force is loaded (right).

First, we investigated the calibration results of the force sensor for the normal and shear direction. Figure 6a describes the relationship between the voltage measured by the photomicrosensor and force measured by the load cell in the indenter for normal direction and shear direction. The red solid line in Figure 6a shows the relationship between the force and voltage measured and the blue dotted line indicates the regression result of the quadratic for the normal and the result of the linear for the shear direction. According to Equation (1), the relationships between the force and voltage were quadratic for the normal direction and linear for the shear direction. From the result of the quadratic regression of the relationship in the normal direction, the coefficient of correlation (R^2^) between the red solid line and blue dotted line was 0.99. From the linear regression result for the shear direction, the R^2^ between the red solid line and blue dotted line was 0.98. These results indicate that the calibration process was the quadratic regression for the normal and linear regression for the direction when converting the voltage value of the developed sensor into the force.

Second, hysteresis is the maximum difference in the estimated forces during loading and unloading [34]:

(2) $$Hysteresis\;\left(\%\right)=\frac{\max\left(F_{loading}-F_{unloading}\right)}{\max\left(F\right)}\times 100$$

where *Floadng* and *Funloading* are the forces during the commercial load cell loading and unloading on the custom sensor, respectively, and max(*F*) is the maximum force measured by the commercial load cell. The loading and unloading processes were repeated 20 times. Figure 6b shows the results of the loading and unloading test. The calculated hysteresis was 2.91 ± 0.30% for the normal direction and 3.48 ± 0.51% for the shear direction.

Third, the repeatability of a sensor was investigated. The repeatability is given by the following:

(3) $$Repeatability\;\left(\%\right)=\frac{1}{N+1}\overset{N}{\underset{r=0}{\sum }}\left[\frac{F_{est}\left(r\right)-\bar{F_{est}}}{\bar{F_{est}}}\right]\times 100$$

where *N* and *Fest* are the number of repetitions and the measured force, respectively [34]. The response of the force sensor during periodic loading and unloading must be examined for the GRF measurement system because the GRF can be loaded on the sensor many times with different periods in daily life. The frequency of the repeatable input was set as the walking frequency (1.5 Hz) [35]. The repeatability was 0.97% in normal loading and 1.15% in shear loading, indicating that the time varying properties for the custom force sensor are relatively negligible. 

Finally, we analyzed how interference effects occur between the normal and shear axes. The cross sensitivity is defined as shown by Equation (4):

(4) $$Cross\;sensitivity\;\left(\%\right)=\frac{1}{N+1}\overset{N}{\underset{n=0}{\sum }}\left[\frac{F_{ref}\left(n\Delta t\right)-F_{est}\left(n\Delta t\right)}{\max\left(F_{shear}\right)}\right]\times 100$$

where *Fref* and *Fest* are the *z*-axis or *x*-axis forces measured by the commercial load cell and the custom force sensor, respectively, and *n*, Δ*t*, and *N* are the data point number, the sampling time, and the total amount of sample data, respectively [24]. As a result, the cross sensitivity was 0.07% during the loading force in the normal direction and 0.19% during the loading force in the shear direction as shown in Figure 6d. These results were comparable to those of commercial multiaxial sensors that included an additional calibration process using a decoupling matrix [36]. Compared with the previous research, the output voltages of the normal direction and shear direction from the custom force sensor were independent of each other, thus the calibration of the custom force sensor was similar to that of two single axis force sensors. 

### 3.2. Experiments for GRF Measurement System

#### 3.2.1. Forefooot Bending Stiffness Test

**Figure 7:**
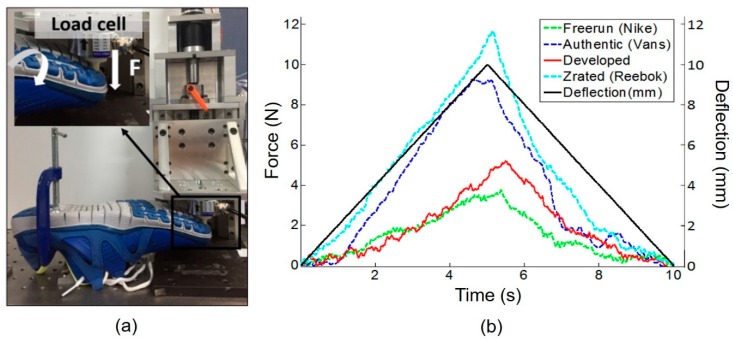
Forefoot bending stiffness test results. (**a**) Experimental setup for the forefoot bending stiffness test. (**b**) Results of the forefoot bending stiffness tests for three commercial shoes with the developed GRF measurement system: Right *y*-axis means the deflection of the load cell and black solid line indicates the imposed deflection of the load cell. Left *y*-axis means the force measured by the load cell and other lines except the black solid line represent the force measured for the commercial shoes.

The developed system was designed for convenient use. In that sense, the forefoot bending stiffness is one of the criteria used to verify convenience. The forefoot bending stiffness is the footwear property that has historically received the least interest compared to other footwear properties, such as the cushioning and thickness of the sole [21,22]. However, the forefoot bending stiffness has drawn considerable attention recently because it is strongly related to athletic performance. Forefoot bending stiffness denotes the stiffness of the MTP joint and is related to the stiffness of the outsole of the shoe [23]. The forefoot bending stiffness of the footwear changes the function of the MTP joint and other mechanics of the lower limb joints, such as the ankle joint torque and the angle change of the joint. An absolute optimal forefoot bending stiffness cannot be easily determined because it depends on the individual person and movement type. We developed the GRF measurement system with an adequate bending stiffness range that overlapped within the range of three commercial running shoes. To measure the forefoot bending stiffness of the MTP joint, a cantilever bending test was conducted with the developed system and three running shoes: Z-Rated (Reebok), Authentic (Vans), and Freerun (Nike) [37]. Each shoe was held in the plate bolted to the optical table, and a C-clamp was used to fix the shoe to the plate. As shown in Figure 7a, vertical force was applied to the forefoot by an indenter, which was used to investigate the sensor characteristics. This force was measured using a commercial load cell (MNC200, CAS, Seoul, South Korea). The three shoe lengths were 270 mm, and the bending point was set to 70 mm from the front end of each shoe. For each trial, the probe with the commercial load cell traveled up and down vertically at a constant speed with a displacement of 10 mm at a speed of 2 mm/s. The vertical force measured by the commercial load cell is plotted with respect to time in Figure 7b. In Figure 7b, the left *y*-axis is the force measured by the load cell in the indenter and the right *y*-axis is the deflection of the load cell which moves up and down vertically. The black solid line indicates the reference deflection of the load cell formed by the motor installed in the indenter. As the deflection increased, the load cell moved down to push the shoes. Other lines indicate the force profiles of the commercial running shoes. The force profile of the developed system fell between those of the Authentic and Freerun shoes, meaning that the forefoot bending degree of the developed system is similar to those of the two commercial shoes when the subject contacts the ground with a pawing motion.

#### 3.2.2. GRF Pattern Comparison with Other Outsole GRF Measurement Systems

**Figure 8:**
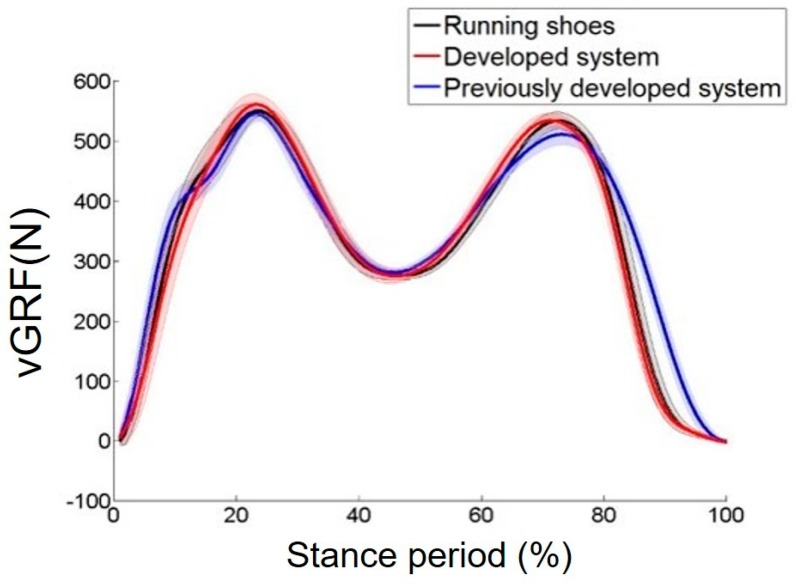
GRF measurement wearing the developed GRF measurement system, previously developed outsole GRF measurement system, and commercial running shoes (Z-Rated, Reebok, Boston, MA, USA).

We investigated how the developed system changed the user’s GRF pattern. Three subjects walked on an instrumented treadmill wearing the developed GRF measurement system, a previously developed outsole GRF measurement system, and the commercial reference running shoes (Z-Rated, Reebok, Boston, MA, USA). The protocol (KH2016-62) was approved by the Institutional Review Board of Korea Advanced Institute of Science and Technology (KAIST). The walking speed was 2.9 km/h. For the previously developed outsole system, we used the outsole GRF measurement system introduced by Gu et al. [24,25]. We used this system as the comparison group because most previously introduced outsole type multiaxial GRF measurement systems share similar forms, with two large GRF measuring sensors installed under the front and rear of the foot [16,17,18,19]. Figure 8 shows the GRF from the three conditions. The GRF patterns appear to be similar, but in the previous developed outsole system, the GRF pattern at a gait cycle of 10% to 15% differed from that measured by the developed system and the reference running shoes. Moreover, the toe off time was longer when wearing the previous outsole system, as shown from the 85% gait cycle to 100% gait cycle. The root mean squared (RMS) differences and standard deviation (SD) between the GRFs measured with the reference running shoe and the developed system was 5.2 ± 1.1 N, and the RMS and SD between the GRFs measured with the reference running shoe and the previously developed outsole system was 19.1 ± 5.3 N. As a result, the GRF profile of the developed system was similar to the GRF profile measured with commercial running shoes as opposed to that measured while wearing the previous system. This result shows that the natural gait could be hindered with the previously developed system, but, the system we developed did not interfere with natural walking.

#### 3.2.3. Walking, Running, and Jumping Experiments 

**Figure 9:**
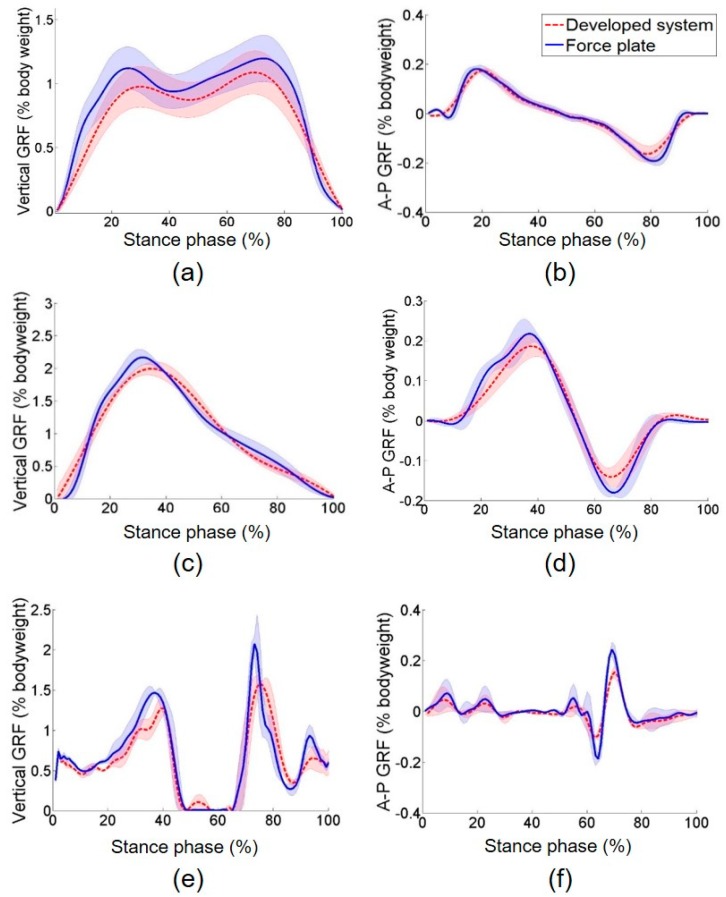
Walking, running, and jumping experimental results. Vertical ground reaction force with the percentage bodyweight of a subject measured wearing the developed GRF measurement system during (**a**) walking, (**c**) running, (**e**) and jumping. Anterior posterior ground reaction force with the percentage bodyweight of a subject measured wearing the developed GRF measurement system during (**b**) walking, (**d**) running, (**f**) and jumping.

**Figure 10:**
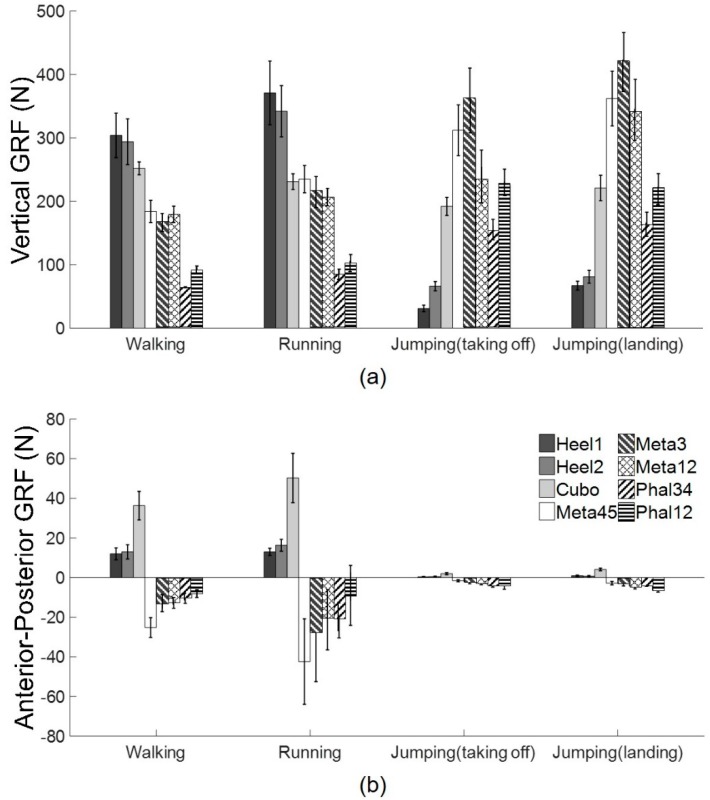
(**a**) Mean and standard deviation of peak vGRF measured by each custom force sensor during walking, running, and jumping (taking off and landing). (**b**) Mean and standard deviation of peak apGRF measured by each custom force sensor during walking, running, and jumping (taking off and landing).

With the developed system, we measured vGRF and apGRF during walking, running, and jumping. To validate the ability of our system to measure the GRF, six subjects (25 ± 1 years, 174.3 ± 2.4 cm, 71.7 ± 4.4 kg, and six males) wearing the developed the GRF measurement system walked, ran, and jumped on an instrumented treadmill (Instrumented Treadmill, Bertec, Columbus, OH, USA). The protocol (KH2016-62) was approved by the Institutional Review Board of Korea Advanced Institute of Science and Technology (KAIST). The instrumented treadmill included a force plate implemented in the treadmill to simultaneously measure the three axis forces and three axis moments. Information for the right foot was acquired to compare the GRF values measured by the developed system and by the force plate. The forces measured by the developed GRF measurement system and the force plate were extracted simultaneously using a data acquisition board (NI PCI-6071E, National Instruments, Austin, TX, USA) with an 800 Hz sampling rate. The speeds of walking and running were 2.9 km/h and 6.9 km/h and 100 steps were extracted for each subject. For the jumping test, the subjects performed a standing vertical jump on the force plate and 30 steps were extracted for validation. A low pass filter was used with the reference cutoff frequency (*fc*) [38]. *fc* was 6 Hz for walking, 10 Hz for running, and 15 Hz for jumping. For comparison, the sum of the forces measured by each sensor was compared with the GRF measured by the force plate. To verify the ability to measure vGRF and apGRF, the normalized root mean squared error (NRMSE) between the GRFs from the force plate and from the developed system was calculated for each subject, as described in Equation (5). Equation (5) can be used to obtain the NRMSE values for each step:

(5) $$NRMSE\;\left(\%\right)=\sqrt{\overset{N}{\underset{n=0}{\sum }}\frac{{\left(F_{sys}\left(i\Delta t\right)-F_{ref}\left(i\Delta t\right)\right)}^{2}}{\frac{F_{ref}\left(i\Delta t\right)}{n}}}\times 100$$

where *F_sys_* and *F_ref_* indicate the GRF values measured by the developed system and the force plate, respectively, and *i*, Δ*t*, and *n* are the data point number, the sampling time, and the total amount of data, respectively. The mean and standard deviation of NRMSE were calculated for all steps, presented in Table 1. To identify the differences in error between the GRF measured by the developed system and force plate during multiple cycles with several trial times, statistical analysis was carried out using the repeated measures ANOVA test (*p* < 0.05). From the statistical analysis, we found that there were no significant differences between the GRF measured by the force plate and the developed system (*p* > 0.05) regardless of the number of cycles for each motion. Figure 9 shows the results for a subject. On average, the error occurred 10.4% for vGRF measurement and 6.8% for apGRF measurement. A limitation was that it could not accurately measure the GRF. To be specific, the magnitude and shape of the active peak can be estimated, but the magnitude and shape of the impact peak were difficult to estimate. This limitation may be overcome by the installation of more sensors in the system or by enlarging the area where the sensor can measure. Although error occurred in the measurement of the overall GRF, this system was able to measure vGRF and apGRF on specific components of the sole as shown in Figure 10, and these values cannot be measured by the existing systems, such as the force plate. Moreover, it has an advantage in measuring two axes’ GRF at the same time unlike the insole type GRF measurement system. The load on the heel was the largest, whereas the load on the toe was the smallest. In addition, the most of the apGRF occurred in the middle of the foot, where sensors were located on the cuboid bone and the lateral side of the metatarsal bone.

## 4. Conclusions

In this work, we proposed a bendable outsole biaxial GRF measurement system. Unlike the previous outsole GRF measurement systems with large force sensors and inflexible outsole construction, in the proposed system, small custom force sensors were placed in the proper positions under a flexible thin sole shoe, and the sensors were fixed with a rubber frame made using a 3D printer to guarantee better flexibility. We measured the forefoot bending stiffness and found that the developed GRF measurement system had flexibility similar to that of regular shoes. Furthermore, we compared the pattern of GRF measured wearing the proposed system, the previously developed outsole GRF measurement system, and commercial running shoes during walking on the force plate. We confirmed that the developed system did not interfere with natural human walking. Finally, we analyzed the GRF measurement performance of the proposed system from the GRF measurement tests with six subjects. The results show that the developed system could satisfactorily measure the GRF during walking, running, and jumping. 

## Figures and Tables

**Table 1:** Normalized root mean square errors (NRMSEs) of the vertical and anterior-posterior ground reaction forces (vGRF and apGRF, respectively) depending on the subjects during walking, running, and jumping (unit in %) (*p*-values < 0.05).

	Walking		Running		Jumping	
	vGRF	apGRF	vGRF	apGRF	vGRF	apGRF
Subject 1	8.6 ± 2.5	4.8 ± 1.8	8.5 ± 1.8	5.1 ± 3.1	12.8 ± 3.0	7.4 ± 3.4
Subject 2	11.8 ± 2.1	7.4 ± 1.5	10.6 ± 2.3	7.9 ± 2.4	11.3 ± 4.2	10.4 ± 4.6
Subject 3	9.1 ± 3.5	6.0 ± 3.0	10.1 ± 2.7	5.7 ± 3.0	14.8 ± 4.0	8.1 ± 3.9
Subject 4	9.6 ± 2.8	5.2 ± 2.1	8.9 ± 1.9	6.7 ± 2.7	11.6 ± 2.5	8.4 ± 2.8
Subject 5	7.1 ± 1.9	4.4 ± 1.9	9.2 ± 2.1	4.9 ± 2.9	12.8 ± 3.1	7.9 ± 1.4
Subject 6	8.4 ± 2.0	4.6 ± 2.0	9.1 ± 2.0	7.8 ± 3.8	13.1 ± 4.4	8.8 ± 3.5
Average	9.1 ± 2.4	5.4 ± 2.1	9.4 ± 2.1	6.4 ± 3.0	12.7 ± 3.5	8.5 ± 3.2
